# The *Trypanosoma brucei* dihydroxyacetonephosphate acyltransferase *Tb*DAT is dispensable for normal growth but important for synthesis of ether glycerophospholipids

**DOI:** 10.1371/journal.pone.0181432

**Published:** 2017-07-17

**Authors:** Rachel Zufferey, Karim Pirani, Melanie Cheung-See-Kit, Sungsu Lee, Tyler A. Williams, Daniel G. Chen, Md. Faruk Hossain

**Affiliations:** 1 Department of Biochemistry, Kansas State University, Manhattan, Kansas, United States of America; 2 Department of Biological Sciences, St. John’s University, Queens, New York, United States of America; Universidade Federal do Rio de Janeiro, BRAZIL

## Abstract

Glycerophospholipids are the most abundant constituents of biological membranes in *Trypanosoma brucei*, which causes sleeping sickness in humans and nagana in cattle. They are essential cellular components that fulfill various important functions beyond their structural role in biological membranes such as in signal transduction, regulation of membrane trafficking or control of cell cycle progression. Our previous studies have established that the glycerol-3-phosphate acyltransferase *Tb*GAT is dispensable for growth, viability, and ester lipid biosynthesis suggesting the existence of another initial acyltransferase(s). This work presents the characterization of the alternative, dihydroxyacetonephosphate acyltransferase *Tb*DAT, which acylates primarily dihydroxyacetonephosphate and prefers palmitoyl-CoA as an acyl-CoA donor. *TbDAT* restores the viability of a yeast double null mutant that lacks glycerol-3-phosphate and dihydroxyacetonephosphate acyltransferase activities. A conditional null mutant of *TbDAT* in *T*. *brucei* procyclic form was created and characterized. *TbDAT* was important for survival during stationary phase and synthesis of ether lipids. In contrast, *Tb*DAT was dispensable for normal growth. Our results show that in *T*. *brucei* procyclic forms i) *Tb*DAT but not *Tb*GAT is the physiologically relevant initial acyltransferase and ii) ether lipid precursors are primarily made by *Tb*DAT.

## Introduction

*Trypanosoma brucei* is a protozoan parasite of the Trypanosomatidae family, which is responsible for important diseases termed sleeping sickness in humans and nagana in domestic animals in Africa. This microorganism alternates between two hosts during its complex digenetic life cycle, the tsetse fly insect vector and the bloodstream of a vertebrate animal.

*T*. *brucei* membranes are made predominantly of glycerophospholipids, which represent 80% of total cellular lipids [1, 2]. The main function of glycerophospholipids is structural by forming the bilayer of the biological membrane. In addition, they fulfill various essential functions in cell cycle progression or in shaping the morphology of organelles such as mitochondria or endoplasmic reticulum; they also regulate signal transduction pathways as second messengers and control membrane trafficking [3–11]. *T*. *brucei* membranes contain the typical phospholipids found in most eukaryotic cells, phosphatidylcholine being the most abundant (PC, 45–60%) followed by phosphatidylethanolamine (PE, 10–20%), phosphatidylinositol (PI, 6–12%), phosphatidylserine (PS, <4%), and cardiolipin (<3%) [2, 10, 11].

Ether glycerophospholipids are a subset of glycerophospholipids that are characterized by the presence of a fatty alcohol instead of a fatty acid at the *sn*-1 position of the glycerol backbone. In *T*. *brucei*, they are primarily found in PE and PS classes [1, 2]. Ether lipids have been shown to be important for the integrity of the endoplasmic reticulum and Golgi apparatus, endocytosis, cholesterol trafficking in mammalian cell lines [12]. While cancer cells overproduce ether lipids for pro-survival purposes, Alzheimer’s disease has been associated with a decline in ether lipid production [13]. Ether glycerophospholipids are also essential for the myelination of nerve cells, development of the brain, eye lens, testes, and for spermatogenesis in mice [14, 15].

The biosynthesis of ether glycerophospholipids initiates with the acylation of dihydroxyacetonephosphate (DHAP) by a DHAP acyltransferase (DHAPAT). The acyl group is then exchanged with a fatty alcohol moiety by the subsequent action of an alkyl-DHAP synthase to yield alkyl-DHAP. The reduction of the latter to alkyl-glycerol-3-phosphate (alkyl-G3P) is executed by an acyl/alkyl-DHAP reductase. All these enzymes are associated with the peroxisomes. The DHAPAT can also participate in the production of ester glycerophospholipids in some organisms [16–18]. In that case, 1-acyl-DHAP is directly converted into 1-acyl-G3P by an acyl/alkyl-DHAP reductase.

The DHAPAT enzyme was purified and characterized originally from mammals (human, rat, guinea pig) as a luminal, membrane associated, peroxisomal enzyme with specificity for DHAP [19]. It interacts directly with the alkyl-DHAP synthase with which it forms a multimeric complex [20]. Mutations in the encoding gene have been associated with a human disease called rhizomelic chondrodysplasia punctate, which is characterized by mental retardation, short stature, and dysmorphic facial appearance [21–23].

In the related parasite *Leishmania major*, the two enzymes *Lm*GAT and *Lm*DAT are the only initial acyltransferases [18, 24, 25]. *Lm*GAT specifically acylates G3P, contributing to ester glycerophospholipid biosynthesis only, and is dispensable for parasite growth and pathogenesis [18]. In contrast, *Lm*DAT preferentially adds acyl groups to DHAP, participates in ester as well as ether glycerophospholipid biosynthesis, which is important for normal growth, survival during stationary phase and virulence [25].

In *T*. *brucei*, DHAPAT, alkyl-DHAP synthase and alkyl/acyl-DHAP reductase activities were found to be associated with peroxisome-like organelles, termed glycosomes in trypanosomatidae [26–29]. Only the gene for the alkyl-DHAP synthase has been identified in this parasite [30]. Furthermore, the G3P acyltransferase (GPAT) *Tb*GAT, which initiates the ester glycerophospholipid biosynthesis is dispensable for viability and glycerophospholipid production, suggesting that *T*. *brucei* possesses an additional initial acyltransferase [31]. This work presents the characterization of the alternative initial acyltransferase *Tb*DAT that begins the ether glycerophospholipid biosynthetic pathway in *T*. *brucei*.

## Results and discussion

### Identification of *Tb*DAT

Our previous studies established that the GPAT *Tb*GAT, which initiates the ester glycerophospholipid biosynthesis, is dispensable for viability and glycerophospholipid production, suggesting that *T*. *brucei* possesses an additional initial acyltransferase(s) [31]. Furthermore, *T*. *brucei* bears a DHAPAT, which may be one of the alternative initial acyltransferase(s), but its molecular identity has not been established [28, 31]. Thus, a DHAPAT ortholog gene was searched in *T*. *brucei* genome using *L*. *major Lm*DAT protein sequence (LmjF.34.1090) as a bait, which lead to the identification of Tb927.4.3160 that was named *TbDAT*. The latter encodes a protein of 1242 amino acids with a calculated molecular mass of 137 kDa. *Tb*DAT is much larger than the orthologs of higher eukaryotes such as mouse or fly [25]. The N-terminal domain does not show any similarity to any known proteins as the leishmanial ortholog while the C-terminal domain contains the four typical conserved motifs that are likely involved in catalysis and substrate recognition [25].

### *T*. *brucei Tb*DAT suppresses the lethal phenotype of a *S*. *cerevisiae* double null mutant lacking endogenous GPAT and DHAPAT activities

**Fig 1:**
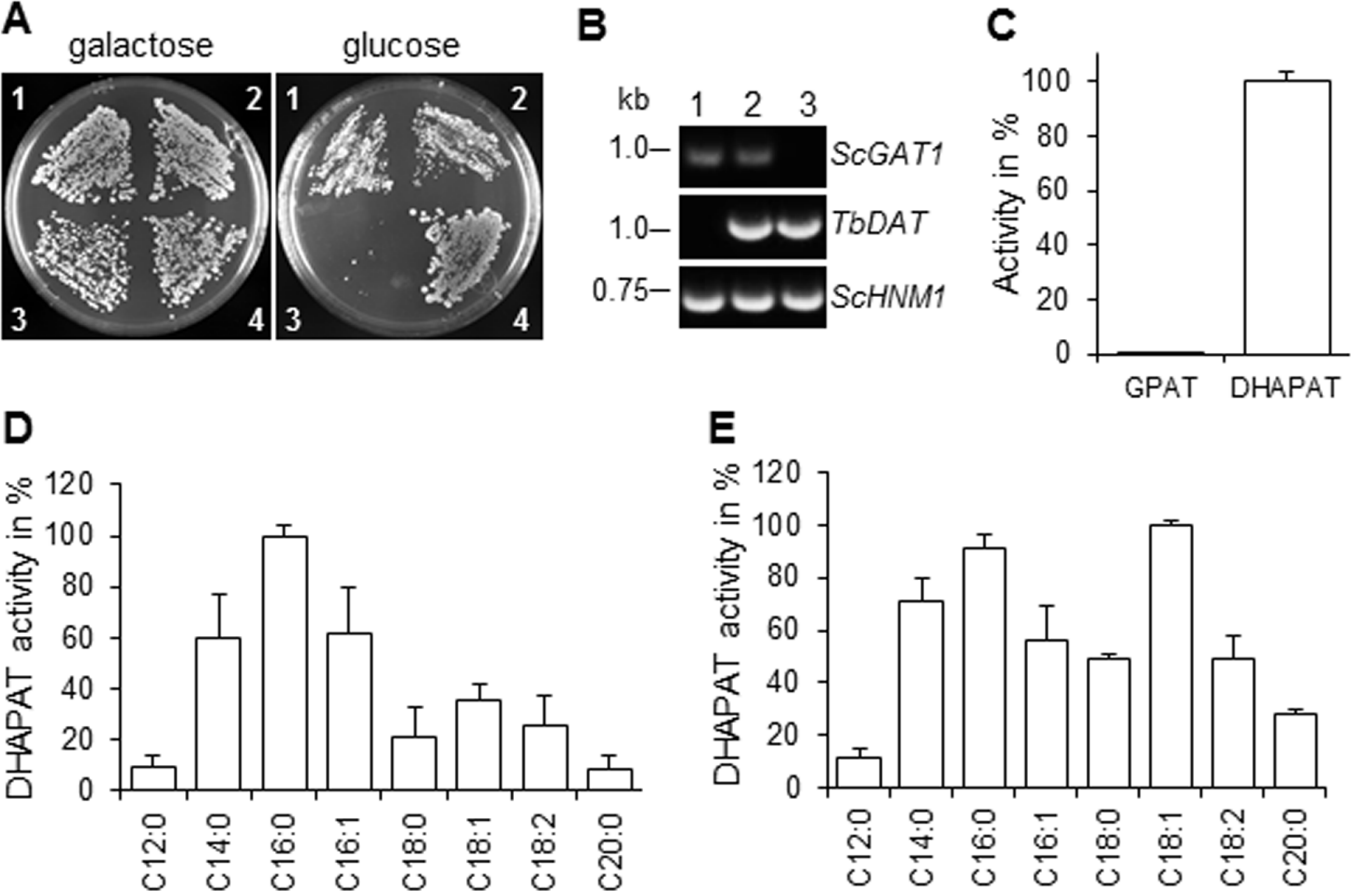
*TbDAT* complements the lethal phenotype of a yeast double null mutant *scgat1Δscgat2Δ*. **(A)** Growth on galactose or glucose-containing medium. 1, *scgat1Δscgat2Δ [GAL1*:*ScGAT1][ADH1*:*TbDAT]* clone 1; 2, *scgat1Δscgat2Δ [GAL1*:*ScGAT1][ADH1*:*TbDAT]* clone 2; 3, *scgat1Δscgat2Δ [GAL1*:*ScGAT1][ADH1]*; 4, *scgat1Δscgat2Δ [GAL1*:*ScGAT1][ADH1*:*LmDAT]* [25]. **(B)** PCR analysis on *S*. *cerevisiae* strains *scgat1Δscgat2Δ [GAL1*:*ScGAT1]*[32] (1), *scgat1Δscgat2Δ [GAL1*:*ScGAT1][ADH1*:*TbDAT]* (2) and *scgat1Δscgat2Δ [ADH1*:*TbDAT]* (3). Primers 652 (5’-ATACGAAGGGCTGTGTAGG-3’) and 653 (5’-TCAACACCGATTTCACCG-3’), 120 (5’-CGGGATCCTCTAGACTACATCCTTGATGCCCGCTTG-3’) and 131 (5’-AGCTAAGATGTTGTGGCTCCGTG-3’), and 654 (5’-CATTGCTTGTCACACTTGG-3’) and 655 (5’- TCACTTCTTTCCCCACGGTAC-3’), were used to amplify *ScGAT1*, *TbDAT* and *ScHNM1* (positive control), respectively. **(C)** GPAT and DHAPAT activities of *scgat1Δscgat2Δ [ADH1*:*TbDAT]*. The GPAT and DHAPAT assays were performed with 0.2 and 1.5 mg proteins derived from whole cell lysates, respectively, and 150 μM palmitoyl-CoA. 100% DHAPAT activity corresponds to 66 pmol/minxmg. **(D)** Fatty acyl-CoA specificity of *Tb*DAT expressed in *scgat1Δscgat2Δ [ADH1*:*TbDAT]*. The values were normalized to the best fatty acyl-CoA donor palmitoyl-CoA. 100% DHAPAT corresponds to 66 pmol/minxmg. **(E)** Fatty acyl-CoA specificity of DHAPAT activity present in *T*. *brucei* whole cell extracts. The values were normalized to the best fatty acyl-CoA donor oleoyl-CoA. 100% DHAPAT activity corresponds to 5.16 nmol/minxmg. (**C**)(**D**)(**E**) The enzymatic assays were carried out at least twice in duplicate and standard deviations are shown.

*S*. *cerevisiae* possesses two initial acyltransferases *Sc*GAT1 and *Sc*GAT2 that have the capability to acylate both DHAP and G3P with similar efficiencies [32, 33]. Single deletion of either gene is not deleterious for life but inactivation of both genes is lethal. Thus, *Tb*DAT was expressed under the control of the constitutive promoter *ADH1* in a *S*. *cerevisiae* conditional double null mutant *scgat1Δscgat2Δ [GAL1*:*ScGAT1]* that synthesizes its endogenous *ScGAT1* in the presence of galactose but not in the presence of glucose [32]. Double transformants *scgat1Δscgat2Δ [GAL1*:*ScGAT1][ADH1]*, *scgat1Δscgat2Δ [GAL1*:*ScGAT1][ADH1*:*TbDAT]* and *scgat1Δscgat2Δ [GAL1*:*ScGAT1][ADH1*:*LmDAT]* [25] gave colonies on galactose containing medium, while *scgat1Δscgat2Δ [GAL1*:*ScGAT1][ADH1]*failed to grow on glucose containing medium as expected (Fig 1A). *TbDAT* conferred survival of the double null mutant *scgat1Δscgat2Δ* on glucose similar to the positive control *scgat1Δscgat2Δ [GAL1*:*ScGAT1][ADH1*:*LmDAT]* [25]. This assay demonstrates that *TbDAT* suppresses the lethal phenotype of a *S*. *cerevisiae* double null mutant lacking both endogenous initial acyltransferases.

### *Tb*DAT restores normal growth, survival in stationary phase and production of normal migrating lipophosphoglycan of the *Leishmania* null mutant *Δlmdat/Δlmdat*

**Fig 2:**
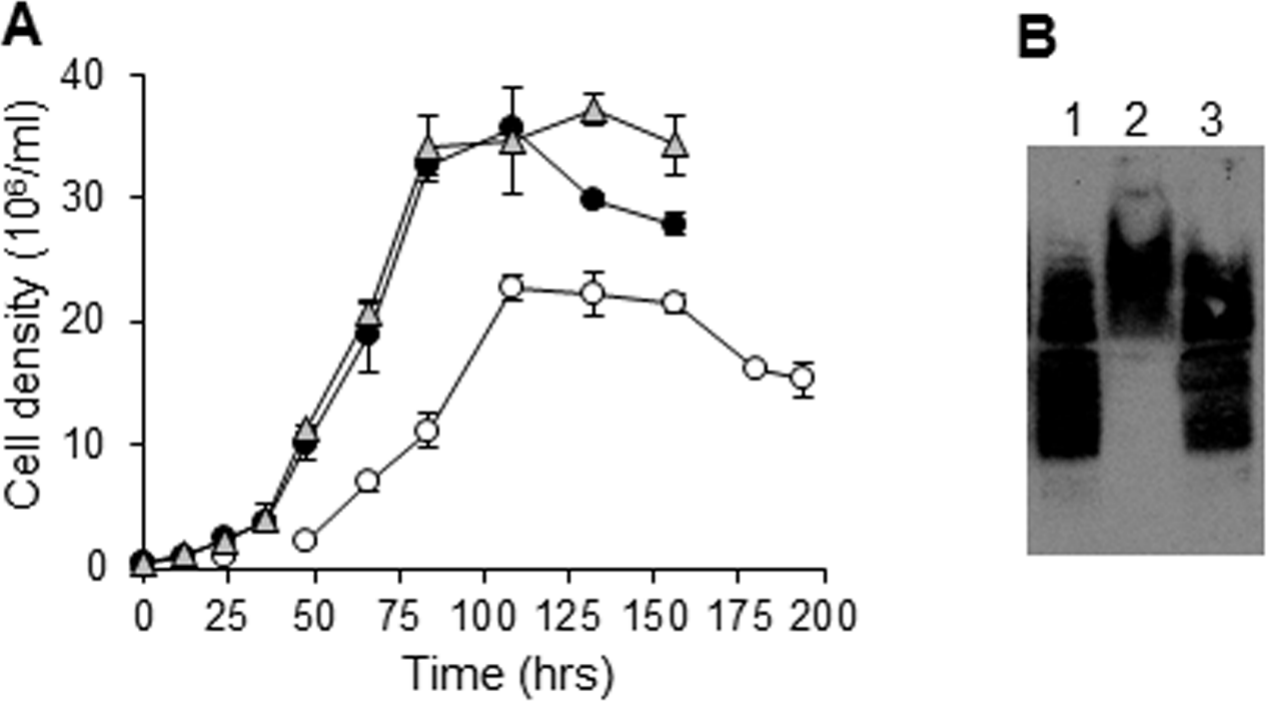
*TbDAT* complements the slow growth phenotype and restores survival during the stationary phase of growth of the *L*. *major* null mutant *Δlmdat/Δlmdat*. **(A)** Growth curves were carried out as described in Methods’ section. White circles, null mutant *Δlmdat/Δlmdat* [25]; black circles, complemented line *Δlmdat/Δlmdat [LmDAT NEO]*; grey circles, *Δlmdat/Δlmdat [TbDAT NEO]*. This assay was performed twice in duplicate and standard deviations are shown. (**B**) Western blot analysis in the presence of lipophosphoglycan specific antibody WIC79.3 [35]. Equivalent of 2x10^6^ cells was loaded in each lane. 1, complemented line *Δlmdat/Δlmdat [LmDAT NEO]*; 2, *Δlmdat/Δlmdat*; 3, *Δlmdat/Δlmdat [TbDAT NEO]*.

The function of *Tb*DAT was further investigated by expressing it in a *L*. *major* null mutant *Δlmdat/Δlmdat*. This strain exhibits a slow growth phenotype, poor survival during the stationary phase of growth and synthesizes an altered form of the ether lipid-anchored virulence factor lipophosphoglycan that migrates slower in a SDS-polyacrylamide gel [25]. Expression of *TbDAT* in the null mutant *Δlmdat/Δlmdat* restored normal growth and survival during the stationary phase of growth (Fig 2A). Furthermore, similar to the complemented strain *Δlmdat/Δlmdat [LmDAT NEO]*, *Δlmdat/Δlmdat [TbDAT NEO]* produced normal forms of lipophosphoglycan, used here as a reporter for ether lipids while the null mutant produced slower migrating lipophosphoglycan (Fig 2B; [34]). These data suggest that *Tb*DAT fulfills similar functions as *Lm*DAT.

### *TbDAT* encodes a DHAPAT enzyme that exhibits preference for palmitoyl-CoA

To assess whether *Tb*DAT acts as a DHAPAT enzyme, the gene was expressed in a constitutive fashion in the *S*. *cerevisiae* double null mutant *scgat1Δscgat2Δ*, which lacks GPAT and DHAPAT activities, in the absence of the *[GAL1*:*ScGAT1]* episome. GPAT and DHAPAT activity assays were then performed. *Tb*DAT acylated DHAP with very high efficiency but not G3P (Fig 1C), demonstrating that DHAP rather than G3P is the preferred substrate of *Tb*DAT.

The fatty acyl-CoA specificity of *Tb*DAT was further investigated. The preferred acyl-CoA donor was palmitoyl-CoA followed by myristoyl-CoA, palmitoleoyl-CoA and oleoyl-CoA (Fig 1D). Lauryl-CoA, stearoyl-CoA, linoleoyl-CoA and eicosanoyl-CoA were the least effective fatty acyl-CoA donors.

The fatty acyl-CoA specificity of the DHAPAT activity present in *T*. *brucei* cell extracts gave a slightly different pattern than that of *Tb*DAT. The best activity was achieved in the presence of oleoyl-CoA and palmitoyl-CoA, followed by myristoyl-CoA, palmitoleoyl-CoA, linoleoyl-CoA, stearoyl-CoA, eicosanoyl-CoA and lastly myristoyl-CoA (Fig 1E). This result suggests that *Tb*DAT is not the only DHAPAT enzyme in this parasite and that another DHAPAT enzyme(s) with different fatty acyl-CoA preference may exist.

### *Tb*DAT localizes to the glycosomes

**Fig 3:**
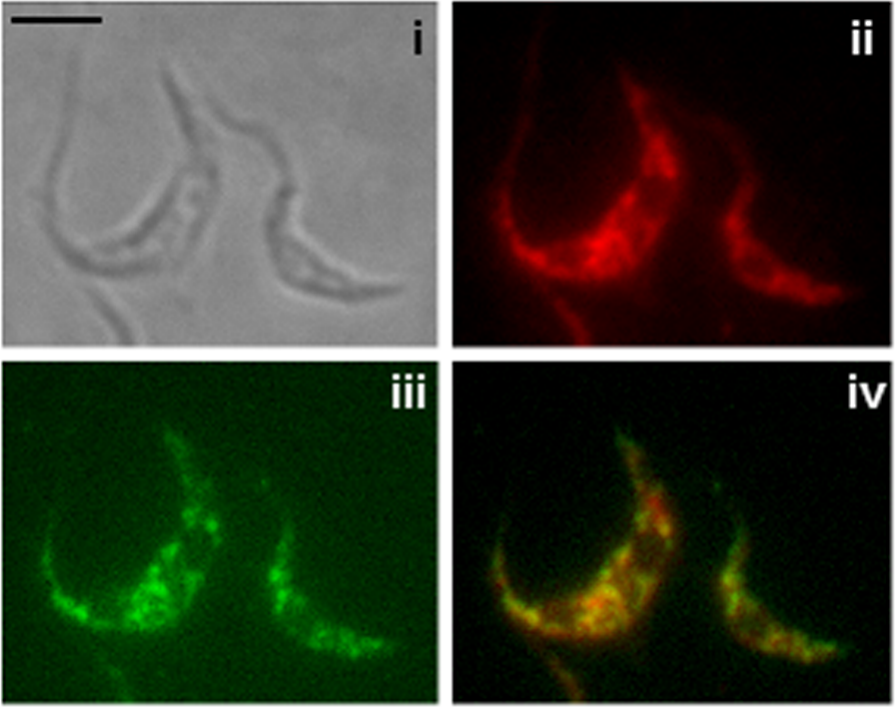
V5:*Tb*DAT localizes to the glycosome. A V5-tagged version of *Tb*DAT was expressed in the null mutant *Δtbdat/Δtbdat* background. Immunofluorescence assay in the presence of anti-V5 monoclonal antibodies (ii) and glycosome specific antiserum [37] (iii). i, DIC; iv, overlap of ii and iii. The bar represents 10 μm.

To determine the subcellular localization of *Tb*DAT, the enzyme was tagged with a V5 epitope at its N-terminus. A C-terminal tag was not attempted due to the presence of a putative peroxisomal targeting tripeptide SRM, suggesting that *Tb*DAT resides in the glycosomes, which are peroxisomal related organelles in trypanosomes [36]. Immunofluorescence assay was carried out in the presence of anti-V5 antibodies, which gave a punctated stain within the parasite cell, a signal that overlapped nicely with that of glycosomes revealed with anti-glycosomal specific antibodies (Fig 3; [37]). This assay demonstrates that *Tb*DAT resides in the glycosomes and is consistent with cell fractionation data established previously by Opperdoes and colleagues [26, 28, 29]. In contrast to other eukaryotes, most of the glycolytic enzymes are compartmentalized in the glycosomes in trypanosomatidae. Thus, the substrate DHAP, resulting from the breakdown of fructose 1,6-bisphosphate by a glycosomal aldolase and triose isomerase, is readily available for the *Tb*DAT enzyme and does not need to be imported into the glycosomes [36, 38, 39]. In contrast, acyl-CoAs need to be transported into the glycosomes by unknown transport proteins as they are produced in the mitochondria by a type II fatty acid synthase complex and in the endoplasmic reticulum by an unusual elongase system [11, 40–42].

### A conditional null mutant of *TbDAT* is viable but survives poorly in the stationary phase of growth

**Fig 4:**
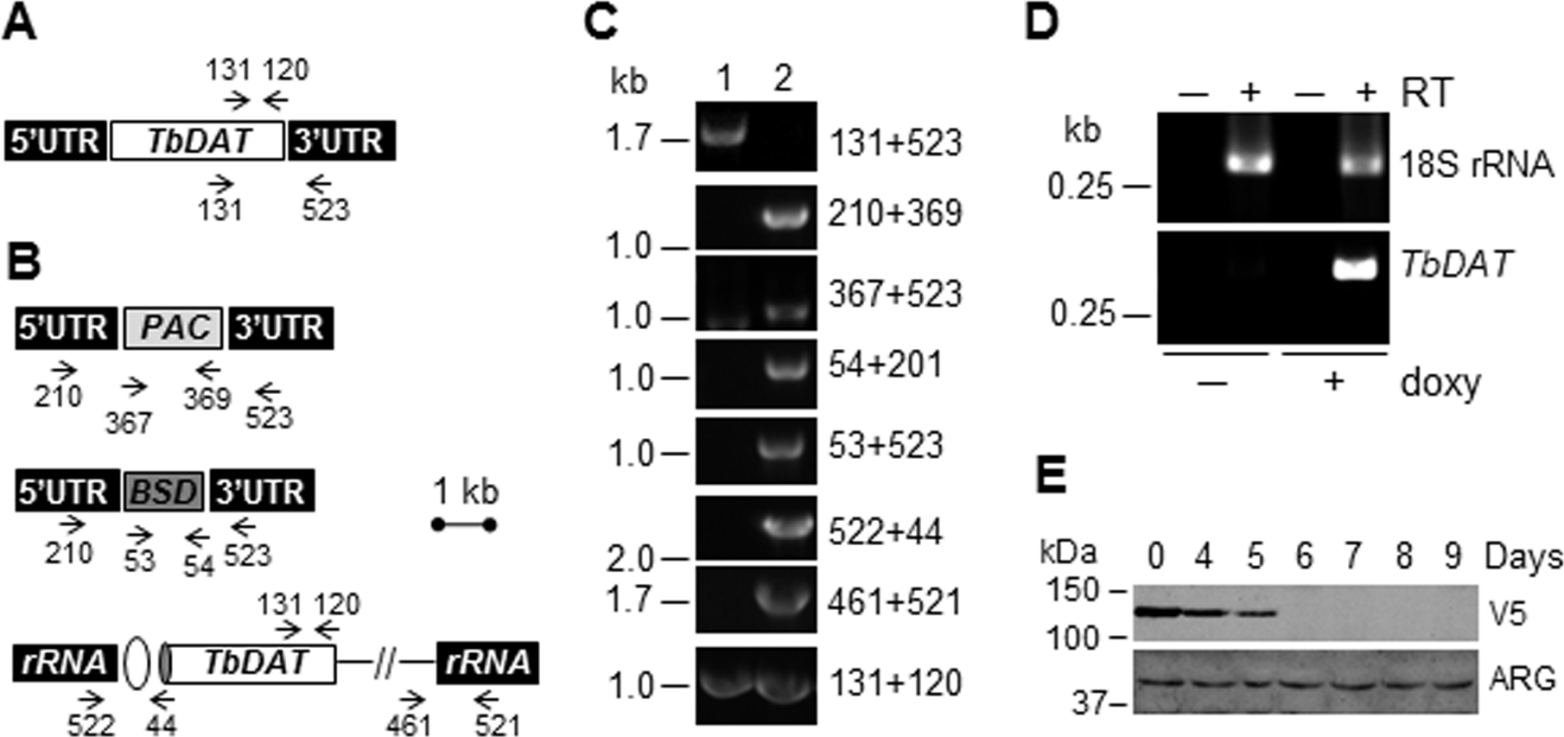
Generation of a conditional null mutant of *TbDAT* in *T*. *brucei*. **(A)** Wild-type organization of the *TbDAT* allele. **(B)** Genotype of the conditional null mutant *Δtbdat/Δtbdat/TetO*:*V5*:*TbDAT* that expresses a V5-tagged (grey oval) version of *Tb*DAT that is regulated by the *TetO* promotor (white oval) and inserted into the intergenic region of the ribosomal RNA locus. Both *TbDAT* genes were replaced with the resistance cassettes blasticidin (*BSD*, dark grey rectangle) and puromycin (*PAC*, light grey rectangle). **(C)** Agarose gel electrophoresis of PCR analysis of the conditional null mutant *Δtbdat/Δtbdat/TetO*:*V5*:*TbDAT*. The position of the primers used here is depicted in (A) and (B). **(D)** RT-PCR analysis of strain *Δtbdat/Δtbdat/TetO*:*V5*:*TbDAT*. RNA was isolated from strain *Δtbdat/Δtbdat/TetO*:*V5*:*TbDAT* grown in the presence (+) or absence (−; 10 days) of doxycycline (doxy) that was (+) or was not (−) subjected to the reverse transcriptase reaction (RT). Amplification of the *18S rRNA* cDNA was used as a positive control. **(E)** Western blot analyses in the presence of anti-V5 (Thermo Fisher Scientific, Waltham, MA) to reveal V5:*Tb*DAT and arginase specific antibodies (ARG; [43]) as a loading control. Approximately 1x10^7^ cell equivalent was loaded in each lane. The protein marker is shown on the left. The Western blot analysis was carried out twice and a representative experiment is shown.

To assess the role of *TbDAT* in parasite’s biology, a tetracycline-dependent conditional null mutant was created by successive transformation and selection in the presence of appropriate antibiotics as described in the Methods’ section. This strategy was chosen as no null mutant could be obtained by traditional successive deletion of the *TbDAT* alleles and RNA interference was unsuccessful. The conditional null mutant bears replacement of both *TbDAT* alleles with antibiotic cassettes *BSD*, that mediates blasticidin resistance, and *PAC*, that relieves puromycin sensitivity, and expresses an N-terminal V5-tagged *Tb*DAT under the control of a tetracycline *TetO* regulated promoter (Fig 4B). The genotype of the conditional null mutant *Δtbdat/Δtbdat*/*TetO*:*V5*:*TbDAT* was verified by PCR using appropriate primers (Fig 4C). Three clones were obtained and showed similar phenotypes. Only one clone is described in details in this work. V5-*Tb*DAT expression is maintained in the presence of doxycycline but it is abolished after 5–6 days in the absence of antibiotic as demonstrated by RT-PCR and Western blot analysis (Fig 4D and 4E). The fact that V5-*Tb*DAT expression took 5–6 days to reach undetectable levels seems to suggest that this enzyme or its mRNA may have a very low turnover or a very long half-life.

**Fig 5:**
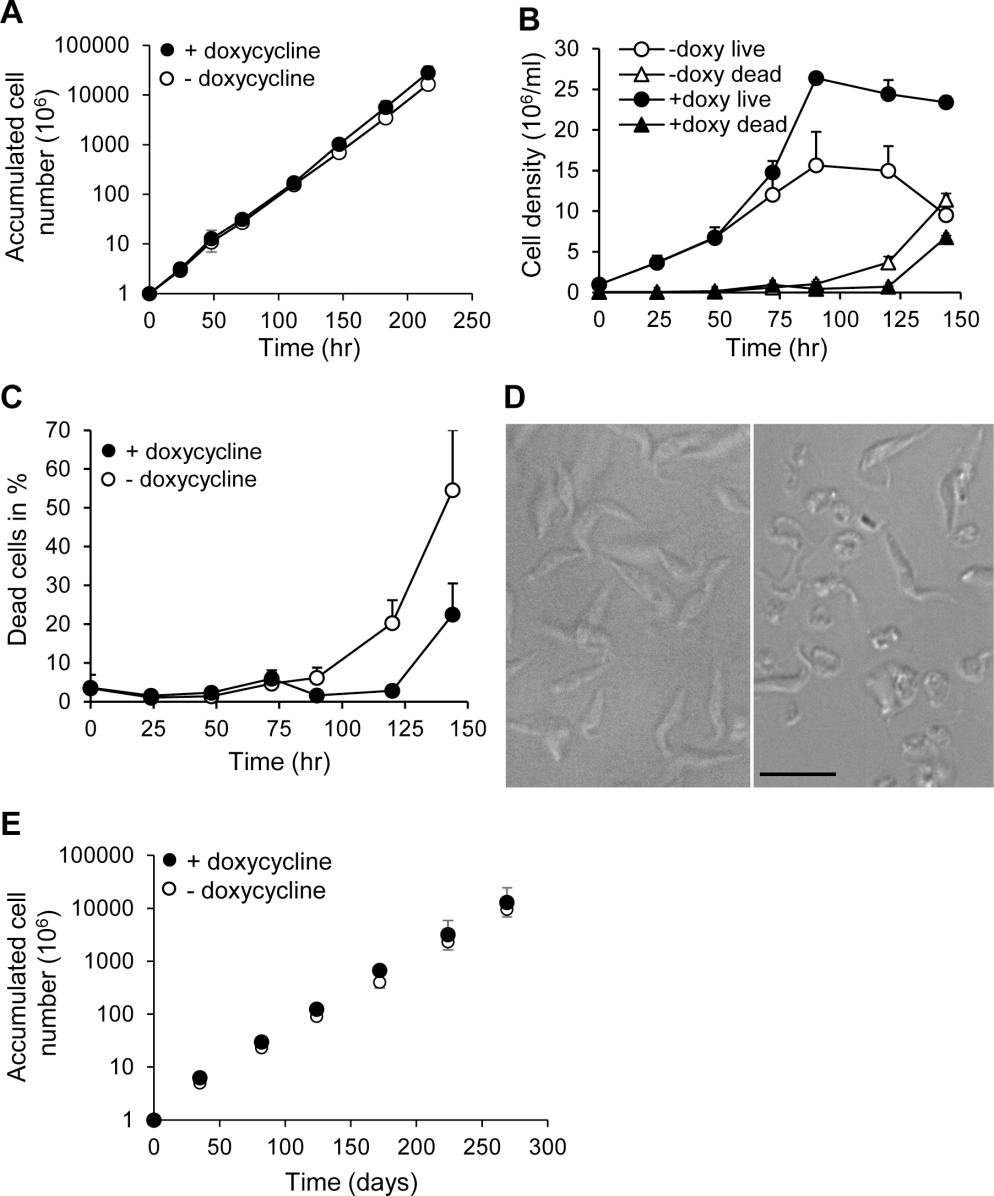
*Tb*DAT is dispensable for normal growth but is important for survival during the stationary phase. **(A)** Growth curves in the presence (black circles) or absence (white circles) of 1 μM doxycycline. (**B**) Growth curves in the presence (black circles, black triangles) or absence (white circles, white triangles) of 1 μM doxycycline. Dead cells were revealed by Zombie green staining. **(C)** Quantification of dead cells expressed in percentage of total cells as a function of time. **(D)** DIC of two days stationary phase of growth cells grown in the presence (left panel) or absence (right panel) of doxycycline. The scale bar represents 10 μm. **(E)** Growth curve of *Δtbdat/Δtbdat/TetO*:*V5*:*TbDAT* grown in the presence (black circles) or absence (white circles) of doxycycline in delipidated medium. (**A**)(**B**)(**C**)(**E**) Growth curves were performed at least twice in duplicate and standard deviations are shown.

The importance of *Tb*DAT in cell viability and growth was then investigated. The conditional null mutant *Δtbdat/Δtbdat*/*TetO*:*V5*:*TbDAT* exhibited similar growth rate in the absence as in the presence of doxycycline (Fig 5A), demonstrating that *Tb*DAT is dispensable for normal growth. This result contrasts with that of *L*. *major* null mutant *Δlmdat/Δlmdat*, which exhibited a twice as long generation time compared to that of the wild type [25]. However, the conditional null mutant reached only half of the wild-type maximal cell density and survived poorly during the stationary phase of growth (Fig 5B and 5C) during which cells become round and necrotic (Fig 5D). The latter phenotype is reminiscent of that of the *L*. *major* null mutant *Δlmdat/Δlmdat* [25].

### A conditional null mutant of *TbDAT* exhibited decreased DHAPAT activity

**Fig 6:**
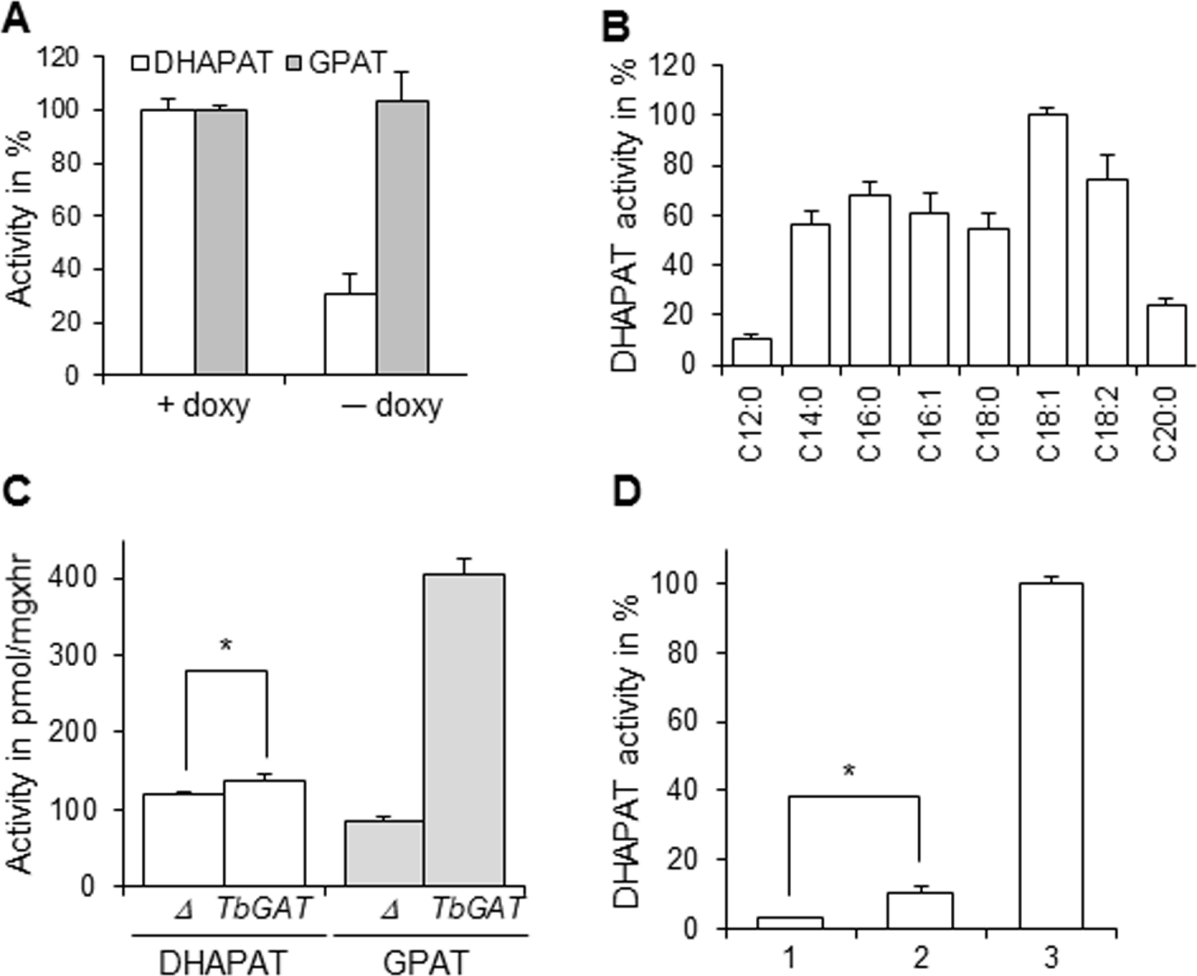
*Tb*DAT is the main DHAPAT enzyme in procyclic trypanosomes. **(A)** The GPAT and DHAPAT assays were performed with 0.2 and 1.5 mg proteins derived from whole cell lysates of cells grown in the presence or absence (10 days) of doxycycline, respectively. 100% of DHAPAT and GPAT activity corresponds to 5.48 nmol/minxmg and 1.31 pmol/minxmg, respectively. **(B)** Fatty acyl-CoA specificity of remaining DHAPAT activity in *Δtbdat/Δtbdat/TetO*:*V5*:*TbDAT* grown in the absence of doxycycline for 10 days. The values were normalized to the best fatty acyl-CoA donor oleoyl-CoA and 100% DHAPAT corresponds to 0.47 nmol/minxmg. **(C)** GPAT and DHAPAT activity quantification of *Δlmgat/Δlmgat* (*Δ*) and *Δlmgat/Δlmgat [TbGAT NEO]* (*TbGAT*) were carried out in the presence of 100 μM and 150 μM oleoyl-CoA, respectively. *, *p* value < 0.05. **(D)** DHAPAT assay with *Δlmdat/Δlmdat* (1, [25]), *Δlmdat/Δlmdat/TbGAT* (2), *and Δlmdat/Δlmdat/LmDAT* (3, [25]). The assay was performed as described in Materials and methods section in the presence of 150 μM oleoyl-CoA and 100% DHAPAT activity corresponds to 1.9 pmol/minxmg. *, *p* value < 0.05. (**A**)(**B**)(**C**)(**D**) The activity assays were carried out at least twice in duplicate and standard deviations are shown.

The DHAPAT activity of *T*. *brucei* conditional null mutant of *TbDAT* was then quantified. This enzymatic activity was present in cells grown in the presence of doxycycline but was diminished in cells grown in its absence by approximately 70% (Fig 6A), indicating that *Tb*DAT is the main DHAPAT and that another enzyme(s) accounts for the remaining activity in procyclic trypanosomes. This result differs from that of *L*. *major*, in which *Lm*DAT was the only DHAPAT enzyme in the parasite [18]. In contrast, GPAT activity levels were comparable in cells grown in the presence or absence of doxycycline, consistent with the fact that *Tb*DAT acylates G3P very inefficiently (Fig 6A).

The fatty acyl-CoA specificity of the remaining DHAPAT activity was then assessed. Best activity was achieved with oleoyl-CoA followed by linoleoyl-CoA, palmitoyl-CoA, myristoyl-CoA, palmitoleoyl-CoA, and stearoyl-CoA. Lauryl-CoA and eicosanoyl-CoA mediated the lowest DHAPAT activity (Fig 6B). This profile is reminiscent of that of the GPAT enzyme *Tb*GAT [31]. Thus, the G3P and DHAP substrate specificity of *Tb*GAT was reinvestigated using *Leishmania* as a heterologous expression system [31]. Expression of *Tb*GAT in the null mutant *Δlmgat/Δlmgat* lead to a five-fold increase in GPAT activity but also to a modest but significant increase in DHAPAT activity, suggesting that *Tb*GAT can contribute to the remaining DHAPAT activity in the *Δtbdat/Δtbdat*/*TetO*:*V5*:*TbDAT* cells grown in the absence of doxycycline (Fig 6C). In addition, *Tb*GAT expression in a *Δlmdat/Δlmdat* mutant, which lacks DHAPAT activity, conferred slight DHAPAT activity as well (Fig 6D). In terms of substrate specificity, *Tb*GAT resembles *S*. *cerevisiae* initial acyltransferases *Sc*GAT1 and *Sc*GAT2 that acylate both G3P and DHAP with similar efficiency [32, 33] and differs from *L*. *major Lm*GAT, which acylates primarily G3P and not DHAP [18]. However, it is not excluded that an additional DHAPAT enzyme may exist in this parasite beside *Tb*DAT and *Tb*GAT. The creation of a double null mutant of *TbDAT* and *TbGAT* will give a definitive answer.

*T*. *brucei* was shown to also scavenge and remodel lipid precursors from the medium as an alternative way to synthesize its cellular glycerophospholipids [2, 8, 9, 44]. Thus, the importance of lipid scavenging was addressed by growing the conditional null mutant in delipidated medium (Fig 5E). Removal of doxycycline did not affect the growth rate, demonstrating that scavenging and remodeling of extracellular lipids are dispensable for the production of parasite’s glycerophospholipids in the absence of *Tb*DAT.

### *Tb*DAT is essential for ether lipid biosynthesis

The consequence of a lack of *TbDAT* expression on ester and ether glycerophospholipid cellular composition was then evaluated. Total cellular lipids were isolated and analyzed by comprehensive mass spectrometry. Levels of ether glycerophospholipids PC, PI, PS, and PE were considerably decreased after doxycycline withdrawal (Table 1). In contrast, amounts of ester lipids were similar when *Tb*DAT is expressed or not expressed, except that PI quantities were slightly but significantly increased in cells grown in the absence of doxycycline, maybe to compensate for the lower levels of the corresponding ether glycerophospholipids. Such a mechanism has been observed in mammalian cells defective in ether lipid metabolism and in *T*. *brucei* depleted for the ethanolamine-specific phosphotransferase gene *TbEPT*, which produced increased levels of ester PE to counteract the lower quantities of ether PE [44, 45].

**Table 1:** Glycerophospholipid quantification.

Strain	WT	*+* doxy	- doxy
ePC	11.44 ± 1.01	9.47 ± 1.38	5.51 ± 0.25*
PC	72.27 ± 6.25	70.51 ± 1.33	75.84 ± 1.33
ePE	2.23 ± 0.75	3.84 ± 0.10	0.45 ± 0.05*
PE	4.81 ± 0.87	6.30 ± 0.07	5.07 ± 0.07
ePI	0.39 ± 0.21	0.27 ± 0.13	0.03 ± 0.00*
PI	6.04 ± 2.35	6.99 ± 0.87	10.90 ± 0.78*
ePS	1.68 ± 0.47	1.68 ± 0.02	0.06 ± 0.03*
PS	1.14 ± 0.16	0.94 ± 0.16	2.13 ± 0.08

Our relative percentages of the individual glycerophospholipid species differ slightly from those reported in [46]. This may be explained by the different lipid extraction protocols applied to isolate total lipids (Folch based *versus* Bligh and Dyer) or the use of a different type of instrumentation. Lastly, the medium composition (especially FBS content) may account for these differences as well.

Lipid species are represented as percentages of total glycerophospholipids. The assay was carried out in quadruplicate and standard deviations are shown. WT, wild type; + doxy, *Δtbdat/Δtbdat/TetO*:*V5*:*TbDAT* grown in the presence of doxycycline;—doxy, *Δtbdat/Δtbdat/TetO*:*V5*:*TbDAT* grown in the absence of doxycycline for 10 days. *, the *p* value resulting from the comparison of *Δtbdat/Δtbdat/TetO*:*V5*:*TbDAT* grown in the presence or absence of doxycycline was < 0.05.

Organellar proteomics have established that *Tb*DAT is also expressed in the bloodstream form of the parasite [29]. In addition, ether lipids were detected in lipidomics analyses of bloodstream form Trypanosomes, suggesting that *Tb*DAT is active in this parasite form as well [1, 46]. Glycolysis is much more active in the bloodstream form of the parasite, thus producing copious amounts of DHAP in the glycosomes to be readily available for *Tb*DAT. It would make sense that the parasite would use *Tb*DAT rather than *Tb*GAT to produce most of its glycerophospholipids, as G3P is a downstream product of DHAP. Thus, *Tb*DAT is likely to be important as well in the bloodstream form of the parasite. The creation of a (conditional) null mutant in the bloodstream form will assess this hypothesis.

**Fig 7:**
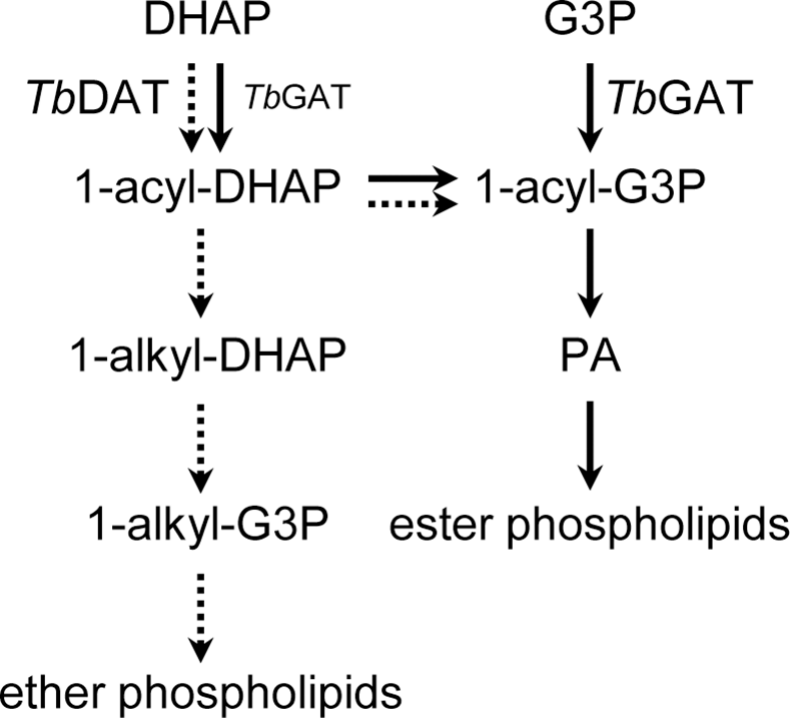
Contribution of *Tb*GAT and *Tb*DAT to glycerophospholipid biosynthesis. *Tb*DAT acylates preferentially DHAP and is involved in the biosynthesis of ether and of ester glycerophospholipids (dotted arrow). In contrast, *Tb*GAT acylates both DHAP and G3P but is implicated mainly in ester glycerophospholipid biosynthesis (solid arrow) [31].

In conclusion, this work shows that DHAPAT activity in the procyclic form of *T*. *brucei* is primarily mediated by *Tb*DAT, whose role is chiefly in ether lipid biosynthesis (Fig 7). In the absence of *Tb*GAT however, *Tb*DAT can also contribute to ester glycerophospholipid production *via* reduction of acyl-DHAP to acyl-G3P [31]. Our studies also suggest that *Tb*GAT is a minor, alternative DHAPAT enzyme, even though it does not contribute to ether but only to ester glycerophospholipid biosynthesis [31].

## Materials and methods

### Strains and growth conditions

The wild-type strain of *T*. *brucei* used in this work is the 29–13 cell line [47]. Parasites were grown in a SD79.3 medium. Delipidated medium was prepared using delipidated fetal bovine serum, which was extracted twice with the same volume of a chloroform/methanol mixture (2:1). The organic solvent’s contaminants were removed by blowing N_2_ (gas) for 3 hr. *Leishmania* mutants *Δlmdat/Δlmdat* and *Δlmgat/Δlmgat*, and complemented lines *Δlmdat/Δlmdat [LmDAT NEO]* and *Δlmgat/Δlmgat [TbGAT NEO]* were described elsewhere and propagated in a liquid M199-based medium in the presence of 20 μg/ml G418, as appropriate [18, 31, 48]. Growth, transformation, and limiting dilution of *Trypanosome* and *Leishmania* parasites were accomplished according to [49]. *T*. *brucei* transformants were selected and/or maintained in the presence of 15 g/ml G418, 25 μg/ml hygromycin, 5 μg/ml blasticidin, 5 μg/ml phleomycin, 1 μg/ml puromycin, 1 μg/ml doxycycline, as appropriate. For growth curves, cells from a mid-log phase culture were diluted to 1x10^6^/ml and cells were counted with a hemocytometer as a function of time.

*Saccharomyces cerevisiae* strains used in this work are *scgat1Δscgat2Δ [GAL1*:*ScGAT1]*, *scgat1Δscgat2Δ [GAL1*:*ScGAT1][ADH1*:*LmDAT]* and *scgat1Δscgat2Δ [GAL1*:*ScGAT1][ADH1]* were described previously [25, 32].

### Plasmids

The plasmid pBEVY-L-TbDAT (Ec312) for expression in *S*. *cerevisiae* was constructed by PCR amplifying the *TbDAT* gene followed by cloning in sense orientation into the BamHI site of pBEVY-L [50]. The same DNA fragment was also inserted in sense orientation into the BamHI site of pXG1a [51] and pLEW100-V5 (Ec877) to give pXG.TbDAT (Ec516) and pLew100-V5:TbDAT (Ec879) for expression in *Leishmania* and *T*. *brucei*, respectively. The episome pLEW100-V5 was created by amplifying the V5 encoding epitope gene by PCR and cloning into the HindIII site of pLEW100 [47].

For inactivation of the *TbDAT* alleles, antibiotic resistance cassettes *BSD* and *PAC* were flanked by approximately 500 bp of *TbDAT UTR* regions to give the plasmids pUC.TbDAT:BSD (Ec383) and pUC.TbGAT:PAC (Ec874), respectively. The antibiotic cassettes were then integrated into the *TbDAT* locus by double crossing over. All PCR-amplified DNAs were verified by sequencing.

### Creation of *S*. *cerevisiae scgat1Δscgat2Δ [ADH1-TbDAT]*

The *S*. *saccharomyces* mutant *scgat1Δscgat2Δ [GAL1*:*ScGAT1* (Sc28 = cmy228) was transformed with pBEVY-L-TbDAT and selected on leucine prototrophy to yield *scgat1Δscgat2Δ [GAL1*:*ScGAT1][ADH1*:*TbDAT]* (Sc31; [32]). Then, 5-fluorouracil was applied to remove the *[GAL1*:*ScGAT1]* episome to give *scgat1Δscgat2Δ [ADH1*:*TbDAT]* (Sc157). The genotype of the resulting cell line was verified by PCR (Fig 1B).

### Creation of the conditional null mutant *Δtbdat/Δtbdat/TetO*:*V5*:*TbDAT*

The conditional null mutant of *TbDAT* was created by the subsequent transformation of the *TbDAT*:*PAC* and the *TbDAT*:*BSD* cassettes that were amplified by PCR using pUC.TbDAT:PAC and pUC.TbDAT:BSD as template, respectively, and oligonucleotides 190 (5’-CCGAAAATGCAAACCACACAC-3’) and 192 (5’-TCCTGTAGGTGACAGATAATGG-3’). The PCR product was directly used for transformation of wild-type procyclic form parasites followed by selection in the presence of puromycin. The resistant heterozygous *TbDAT*:*PAC*/*TbDAT* clone was verified by PCR and then subjected to a second round of transformation with pLew100-V5:TbDAT, which was previously linearized with NotI, to give phleomycin resistant *TbDAT*:*PAC*/*TbDAT/TetO*:*V5*:*TbDAT* cells. Lastly, the latter strain was transformed with the *TbDAT*:*BSD* cassette and parasites resistant to blasticidin, phleomycin and hygromycin were selected. Also, a concentration of 1 μg/mg of doxycycline was applied to maintain *V5*:*TbDAT* expression as needed. The proper chromosomal integrations were all verified by PCR (Fig 4C).

### Reverse transcription (RT) PCR

RNA was purified from mid-log phase parasites with Trizol (Thermo Fisher Scientific, Waltham, MA) as described by the manufacturer’s protocol. DNA contaminant was degraded using a DNA removal kit and RT reaction was carried out by random priming using the SuperScript III reverse transcriptase, both from Thermo Fisher Scientific (Waltham, MA). PCR was carried out with primers 138 (‘5-ACCCGGGCTCACAGAAGAG-3’) and 328 (‘5 CTCTAGACCCATCACCAGGAGCAAATAG-3’) specific to *TbDAT*, or oligonucleotides 539 (5’-ACGCCAAGCTAATACATGAACC-3’) and 540 (5’-TATTTCTCAGGCTCCCTCTCC-3’) specific to *18S rRNA* gene using the Titanium Taq polymerase (Clontech Laboratories Inc., Mountain View, CA).

### Enzymatic assays

*Leishmania*, *S*. *cerevisiae* and *Trypanosoma* whole cell extracts were prepared as described previously [25]. Protein concentration was determined by the bicinchoninic acid assay using bovine serum albumin as a standard.

DHAPAT and GPAT activity assays were performed as described in [18, 25, 31]. Briefly, GPAT assay was performed in a buffer system containing 20 mM TrisHCl pH7.5, 4 mM NaF, 1 mM DTT, 2 mM MgCl_2_, 1 mg/ml fatty acid free BSA, 100 μM fatty acyl-CoA, 0.4 mM G3P (including 5.7 μM of [U^14^C]L-G3P; specific activity of 25 mCi/mmol; MP Biomedicals, LLC, Santa Ana, CA), and 200 μg of protein extract in a total volume of 200 μl. The reaction was incubated at 30°C and stopped after10 min with 700 μl of 1% HClO_4_. The products were extracted with 2 ml of a methanol:chloroform (1:1) mixture and the lower, organic phase was washed with 700 μl of 1% HClO_4_. The organic phase was air dried and the radioactivity was quantified with a scintillation counter.

DHAPAT activity was assessed by measuring the acylation rate of DHAP based on a protocol established by Bates and Saggerson [25, 31, 52]. First, DHAP was produced by catabolism of fructose 1,6-bisphosphate by the enzymes triose isomerase (28 U; Millipore-Sigma, St Louis, MO) and aldolase (0.44 U; Millipore-Sigma, St Louis, MO) in a buffer system containing 50 mM TrisHCl pH7.5, 0.12 M KCl, 1 mM NaF, 4 mM MgCl_2_, 2 mg/ml fatty acid free BSA, 150 μM fatty acyl-CoA, and 0.5 mM fructose 1,6-bisphosphate (including 0.13 µM of [U-^14^C]D-fructose 1,6-bisphosphate; specific activity of 295 mCi/mmol; MP Biomedicals, LLC, Santa Ana, CA). The assay was incubated at 30°C for 15 min after which 0.1% CHAPS and 1.5 mg of cell extracts were added. The reaction was stopped after 10 min by adding 400 μl of 1% HClO_4_. The samples were then extracted as described above for the GPAT assay. Both enzymatic assays were performed at least twice in duplicate. The incorporated radioactivity was quantified by liquid scintillation counting.

### Electrophoresis and Western blot analysis

Proteins were separated by SDS-PAGE (7.5–12.5%/4%) and subjected to Western blot analyses in the presence of antisera specific to the arginase [43], GPEET procyclin (5H3, [53]) or of monoclonal antibodies against the V5 epitope (Thermo Fisher Scientific, Waltham, MA) or lipophosphoglycan (WIC79.3, [35]) according to [25].

### Lipid analysis

Parasites were grown in quadruplicate cultures to late log phase and washed three times with cold PBS. Total cellular lipids were purified according to Schneiter *et al*. [54]. Lipids were profiled by electrospray ionization tandem mass spectrometry (ESI-MS/MS) using the method described by [34], except that internal standards were (with some small variation in amounts in different batches of internal standards): 0.6 nmol di12:0-PC, 0.6 nmol di24:1-PC, 0.6 nmol 13:0-lysoPC, 0.6 nmol 19:0-lysoPC, 0.3 nmol di12:0-PE, 0.3 nmol di23:0-PE, 0.3 nmol 14:0-lysoPE, 0.3 nmol 18:0-lysoPE, 0.3 nmol 14:0-lysophosphatidylglycerol (lysoPG), 0.3 nmol 18:0-lysoPG, 0.3 nmol di14:0-PA, 0.3 nmol di20:0 (phytanoyl)-PA, 0.2 nmol di14:0-PS, 0.2 nmol di20:0(phytanoyl)-PS, 0.23 nmol 16:0–18:0-PI, 0.3 nmol di14:0-PG, and 0.3 nmol di20:0(phytanoyl)-PG. In addition to the scans previously described [34], a scan for PG as [M+NH_4_]^+^ in the positive mode with NL 189.0 was performed with collision energy of 20 V, declustering potential of 100 V, and exit potential of 14 V. Ether-linked (alk(en)yl, acyl) lipids were quantified in comparison to the diacyl compounds with the same head groups without correction for response factors for these compounds as compared to their diacyl analogs.

### Immunofluorescence assay

Immunofluorescence assay was carried out as described previously [31]. The monoclonal antibody specific to V5 (Thermo Fisher Scientific, Waltham, MA, #R96025) and glycosomes were used at a 1:500 and 1:100 dilution, respectively [37, 53].

For viability assay, *T*. *brucei* cells were labeled with the Zombie Green Fixable Viability Kit (BioLegend, San Diego, CA) and washed as stated by the manufacturer’s instructions. Cells were then fixed for 10 min in the presence of 4% paraformaldehyde in PBS and mounted on poly-lysine coated cover slips. Green and red cells were visualized with a fluorescence microscope and quantified with a hemocytometer. At least 200 cells were counted. This assay was performed twice in duplicate.

### Statistical analyses

Data are represented as mean ± SE. The significance of means’ differences was calculated by two-tailed paired t-test using Microsoft Excel data analysis. Differences were considered significant when the *p*-value was < 0.05.
